# Male‐specific mortality biases secondary sex ratio in Eurasian tree sparrows *Passer montanus*

**DOI:** 10.1002/ece3.3575

**Published:** 2017-11-06

**Authors:** Takahiro Kato, Shin Matsui, Yohey Terai, Hideyuki Tanabe, Sayaka Hashima, Satoe Kasahara, Gen Morimoto, Osamu K. Mikami, Keisuke Ueda, Nobuyuki Kutsukake

**Affiliations:** ^1^ Department of Evolutionary Studies of Biosystems School of Advanced Sciences SOKENDAI (The Graduate University for Advanced Studies) Hayama Japan; ^2^ Department of Biology School of Biological Sciences Tokai Univerrsity Sapporo Japan; ^3^ Department of Life Science Graduate School of Engineering Science Akita University Akita Japan; ^4^ Faculty of Agriculture and Life Science Hirosaki University Hirosaki Japan; ^5^ Division of Avian Conservation Yamashina Institute for Ornithology Abiko City Japan; ^6^ Wildlife Conservation Center Department of Life Sciences Toho University Chiba Japan; ^7^ Department of International and Regional Studies Faculty of Education Hokkaido University of Education Hakodate Japan; ^8^ Rikkyo University Saitama Japan

**Keywords:** Eurasian tree sparrow, fertility, *Passer montanus*, primary sex ratio, secondary sex ratio, sex‐specific mortality

## Abstract

Sex allocation theory predicts that parents bias the offspring sex ratio strategically. In avian species, the offspring sex ratio can be biased at multiple growth stages, although the mechanisms are not well known. It is crucial to reveal a cause and timing of biased offspring sex ratio. We investigated (i) offspring sex ratio at multiple growth stages, from laying to fledging; and (ii) the stage at which offspring sex ratio became biased; and (iii) the cause of biased offspring sex ratio in Eurasian tree sparrows *Passer montanus*. Sex determination of 218 offspring, including hatchlings and unhatched eggs from 41 clutches, suggested that the offspring sex ratio was not biased at the egg‐laying stage but was significantly female‐biased after the laying stage due to higher mortality of male embryos. Half of the unhatched eggs showed no sign of embryo development (37/74, 50.00%), and most undeveloped eggs were male (36/37, 97.30%). Additional experiments using an incubator suggested that the cause of embryo developmental failure was a lack of developmental ability within the egg, rather than a failure of incubation. This study highlights the importance of clarifying offspring sex ratio at multiple stages and suggests that offspring sex ratio is adjusted after fertilization.

## 1 INTRODUCTION

Sex allocation theory predicts that parents bias offspring sex ratio strategically. Fisher (1930) proposed that when the payoffs from producing male or female offspring are the same, the offspring sex ratio is equal. Under natural conditions, environmental heterogeneity (e.g., spatial structure, habitat quality, resource abundance, population density) ensures that individuals receive differential payoffs depending on their sex (Charnov, 1993). Previous studies have also highlighted the importance of competitive or cooperative interactions among siblings as factors contributing to differential payoffs (e.g., local resource competition: Clark, 1978; local mate competition: Hamilton, 1967; Werren, 1983; local resource enhancement: Komdeur, Daan, Tinbergen, & Mateman, 1997; Pen & Weissing, 2000). Female parents may facultatively bias the offspring sex ratio when the social context or continuously changing environments affect the fitness of one sex more strongly than the other (e.g., parental condition: Trivers & Willard, 1973; Clutton‐Brock, Albon, & Guinness, 1984; Cockburn, Legge, & Double, 2002; mate attractiveness: Sheldon, Andersson, Griffith, Örnborg, & Sendecka, 1999).

In vertebrates with chromosomal sex determination (e.g., birds, mammals), the proximate mechanisms behind biased sex ratios are not well known (Rutkowska and Badyaev, 2008; Tagirov and Rutkowska, 2013). To understand the mechanisms biasing offspring sex ratio, it is crucial to investigate successive changes in the sex ratio over different developmental stages. In avian species, the sex ratio can change at both the primary (fertilization) and secondary (embryo development, hatching, and fledging) stages. Most research on wild populations of birds has focused on the secondary sex ratio (after egg laying). In birds, the physiological state of the female parent can influence the primary sex ratios (Navara, 2013). The secondary sex ratio can become skewed from the primary sex ratio following sex‐specific mortality (SSM) of offspring. Previous studies showed that one of the causes of SSM was steroid hormones (Cichoń, Sendecka, & Gustafsson, 2005; Love, Chin, Wynne‐Edwards, & Williams, 2005; Navara, 2013; Pérez, Velando, & Domínguez, 2006; Rubolini, Romano, Martinelli, & Saino, 2006; Rutkowska & Cichoń, 2006; Svensson, Rintamäki, Birkhead, Griffith, & Lundberg, 2007; von Engelhardt, Dijkstra, Daan, & Groothuis, 2004; Wu et al., 2012). Additionally, at behavioral level, temperature during embryonic development, regulated by parental incubation, reportedly causes SSM of embryos (DuRant et al., 2016; Eiby, Wilmer, & Booth, 2008). Even after the hatching stage, it has been reported that sexual dimorphism of body size caused SSM (larger‐sized sex have higher mortality: Benito & González‐Solís, 2007; Lee, Hwang, Lee, & Choe, 2010; smaller‐sized sex have higher mortality: Eberhart‐Phillips et al., 2017). Despite this, the specific mechanisms underlying the change in bias from primary to secondary sex ratio remain largely unknown.

Although SSM of offspring occurs in some other taxa (e.g., mammals: Baxter, Jarvis, Palarea‐Albaladejo, & Edwards, 2012; fish: Morán, Labbé, & Garcia de Leaniz, 2016; insects: House, Simmons, Kotiaho, Tomkins, & Hunt, 2010; Lachowsky & Reid, 2014), it remains unclear whether SSM is adaptive for parents. SSM as a means of sex ratio adjustment is puzzling, as female parents incur substantial costs of investment such as egg production. Nonetheless, Alonso‐Alvarez (2006) suggested that SSM might be adaptive in avian species laying large numbers of eggs because the relative cost per offspring lost is small.

Avian species in the genus *Passer* are an ideal system to investigate SSM and the mechanisms that affect the primary sex ratio. In passerine birds, the percentage of hatching success is approximately 90% (Morrow, Arnqvist, & Pitcher, 2002). However, in several species of *Passer* birds, this percentage is much lower than in other species [e.g., 65% in Eurasian tree sparrows, *Passer montanus* (Svensson et al., 2007); 56% in house sparrows, *Passer domesticus* (Aslan, Yavuz, & Erdogan, 2005); and 67% in Spanish sparrows, *Passer hispaniolensis* (Marques, 2003)], although there is large variation among populations. In Eurasian tree sparrows, males have higher mortality than females at the embryonic developmental stage (Svensson et al., 2007). Their mean clutch size is not large, approximately 5 per breeding attempt, but they nest 3 or 4 times and lay many eggs in a breeding season (Kato personal observation). Researchers sometimes assume that unhatched eggs were unfertilized; however, in another study of *P. montanus*, most eggs were fertilized, but the germinal disk in unhatched eggs did not show normal embryo development (Birkhead, Hall, Schut, & Hemmings, 2008). Svensson et al. (2007) determined the sex only of developed unhatched offspring and did not investigate the sex of embryos that showed no sign of development. As mentioned, previous studies reported that SSM of embryos might be caused by two factors. One is the effect of substances in the yolk, such as steroid hormones (Love et al., 2005; Navara, 2013; Rubolini et al., 2006; Rutkowska & Cichoń, 2006; von Engelhardt et al., 2004), and the other is the effect of parental incubation (DuRant et al., 2016; Eiby et al., 2008). Thus, these physiological and behavioral factors can change offspring sex ratio, yet these possibilities have never been directly tested.

In this study, we aimed to reveal (i) offspring sex ratio from egg laying to fledging, (ii) the stage at which the offspring sex ratio became biased, and iii) the cause of biased offspring sex ratio in Eurasian tree sparrows, *P. montanus*. We collected DNA samples from offspring in clutches of wild nests and determined the primary sex ratio (at egg laying, as a proxy for the fertilization stage) and secondary sex ratios at three stages (at embryo development, hatching, and fledging). Moreover, we conducted an experiment to determine whether embryo SSM is caused by lack of development ability or inadequate parental incubation, and investigated egg fertilization.

## 2 METHODS

### 2.1 Fieldwork and data collection

We conducted the study in Ogata village, Akita prefecture, Japan (N40°00′00, E140°00′00), from April to September in 2013, 2015, and 2016. We attached a total of 163 nest boxes in 2013, 127 in 2015, and 137 in 2016 to pine trees (*Pinus thunbergii*) in 7 km of windbreak forest and to 2 warehouses. We recorded breeding parameters (clutch size, number of eggs with developed embryos, number of hatched eggs, and number of fledglings) of Eurasian tree sparrows that nested in the nest boxes. Eurasian tree sparrows laid 1 egg per day until they completed their clutch and incubated their clutch for 12 days until the eggs hatched. We observed embryo development by candling eggs with a torch on the second day after egg laying had finished. We collected dead embryos, dead nestlings, and blood samples from nestlings to determine the sex of offspring. We preserved all samples in 99.5% ethanol at −20°C until sex determination.

### 2.2 Investigation of fertilization of undeveloped embryos

We investigated the fertilization of undeveloped embryos following the methods of Birkhead et al. (2008) and Aslam et al. (2012) in 2016. We collected the germinal disks from 28 undeveloped eggs on the second day after egg laying had finished in 2016. We examined embryo development by candling eggs with a torch. We stained nuclear DNA from the germinal disk (Hoechst 33342) and used fluorescent microscopy to investigate whether the cells in the germinal disk had developed (see Data S1 for details). If the eggs were developing normally, they contained thousands of stained nuclei.

### 2.3 Sex determination

We extracted DNA from the blood of 129 nestlings, the dead bodies of 7 nestlings, 37 embryos that failed to hatch, and 45 germinal disks that failed to develop into embryos (a total of 218 samples from 41 clutches sampled in 2015) using the DNeasy Blood & Tissue Kit (QIAGEN, Tokyo, Japan). We determined the sex of offspring using PCR amplification (2550F–2718R primers) of *CHD1* genes located on the Z and W sex chromosomes (Fridolfsson & Ellegren, 1999; Griffiths, Double, Orr, & Dawson, 1998). A 25‐μl reaction mixture contained 20 ng of DNA (0.5 μl), distilled water (18.90 μl), 0.5 mmol/L primers (0.5 μl each), 1× Taq Buffer (2.5 μl, TaKaRa, Shiga, Japan), 2.5 mmol/L dNTPs (2.0 μl), and Taq polymerase (0.1 μl, 1 units, TaKaRa, Shiga, Japan). The PCR program was hot‐started at 94°C, followed by 35 cycles of 95°C for 30 s, annealing at 48°C for 30 s, and extension at 72°C for 40 s. We determined the sex of offspring from the difference in length of two types of bands; 450 bp was associated with the W‐chromosome, and 600 bp was associated with the Z‐chromosome.

### 2.4 The causes of embryo development failure: experimental design and predictions

We tested whether the cause of embryo development failure was inadequate parental incubation or lack of developmental ability in the egg. In order to separate these possibilities, we conducted the following experiment from 29 March to 9 August 2013. First, on the day when the third egg was laid, we collected one egg randomly from each of 30 clutches. Collected eggs had not been incubated by the parents, as Eurasian tree sparrows begin incubation after the fourth egg is laid (Kato personal observation). We kept the collected eggs in an incubator at 39°C for 72 hr and turned eggs every 60 min using an automatic program. Because we could confirm that some eggs used in this experiment and returned to nests developed normally, indicating that our incubator experiment did not hinder the normal development, we assumed that an incubator would provide optimal conditions for male and female embryo development. We also recorded the percentage of development of the original clutch from which each experimental egg was taken on the second day after egg laying had finished. We predicted that if the cause of SSM was inadequate parental incubation, the collected eggs in the incubator should contain developed embryos at a constant probability, irrespective of the percentage of embryo development in the original clutch from which the egg was removed, as the incubator provides a constant environment. Conversely, we predicted that if the cause of SSM was lack of developmental ability in the egg, collected eggs should show embryo development in the same probability of embryo development as eggs in the original clutch.

### 2.5 Statistical analyses

We conducted all analyses in R 3.3.1 (R Development Core Team (2016)) using generalized linear mixed models (GLMMs) or generalized linear models (GLMs) using “glmer” with package “lme4” and a binomial error structure with a logit link function. We selected the final models by eliminating nonsignificant variables using a likelihood ratio test, with the alpha level set to 0.05. We analyzed whether offspring survived (1) or not (0) at three different stages (development, hatching, and fledging) as the response variable in separate GLMMs. The predictor variable was the sex of offspring (female was input as “0,” male was input as “1”), and the day that egg laying of each clutch started was recorded to track seasonality, because SSM can vary over the breeding season (e.g., Székely, Cuthill, Yezerinac, Griffiths, & János, 2004). Nest box identity was included as a random variable. This analysis used data from 218 eggs (from 41 clutches) laid in 2015, in which we determined offspring sex ratio at laying, embryo development, hatching, and fledging.

To test the cause of embryo development failure, we analyzed the association between the success of embryo development of collected eggs and the percentage of embryo development in the original clutch. This analysis used a GLM in which the response variable was whether a collected egg developed (1) or not (0), judged by candling eggs with a torch. The predictor variables were the percentage of embryo development in the original clutch, the day that egg laying of each clutch started (seasonality), and egg‐laying order.

## 3 RESULTS

### 3.1 Fertility of undeveloped eggs

**Figure 1:**
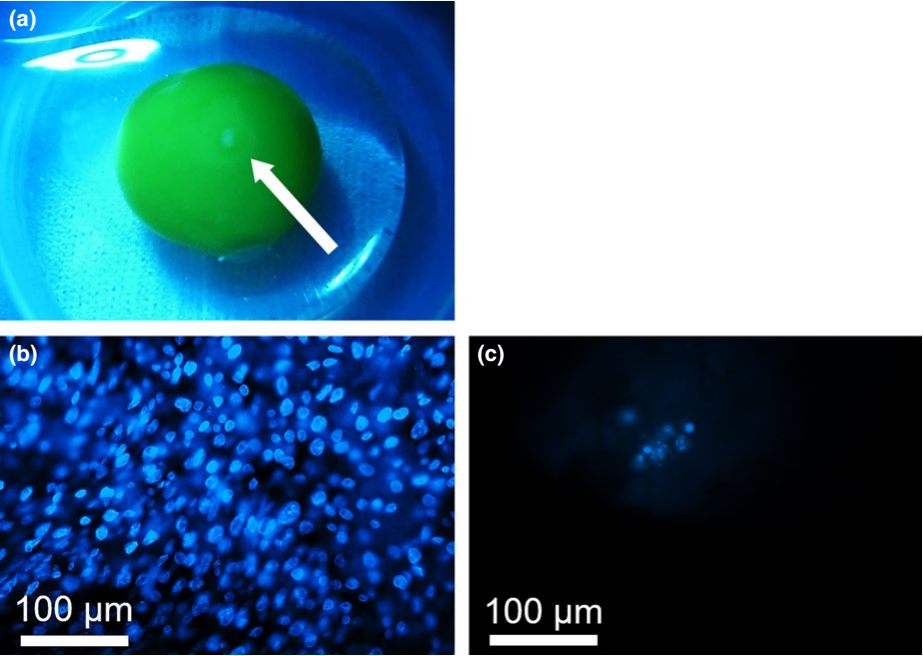
(a) The inner tissue of an undeveloped egg. A white patch in the center of the yolk (indicated by an arrow) is the germinal disk. (b, c) Nuclei were stained with Hoechst 33342, colored blue. A large number of nuclei were observed in fertilized germinal disks after normal development (b), whereas a small number of nuclei were observed in the germinal disks that were fertilized but stopped development at around the 8‐cell stage (c)

We found that 20.64% of all eggs did not show any sign of embryo development (173/218 eggs, Figure 1a, Table 1a). Of the eggs showing embryo development, 78.61% successfully hatched (136/173 embryos, Table 1a). Subsequently, 94.85% of hatchlings successfully fledged (129/136 hatchlings, Table 1a).

**Table 1:** (a) The change in offspring sex ratio from primary (laying) to secondary (embryo development, hatching, fledging) stage. Each *p*‐value was calculated using the binomial test. (b) Number and sex of dead offspring from the preceding developmental stage. Figures in parentheses show the percentage of mortality from the preceding developmental stage. Each *p*‐value was calculated using the proportional test to examine the difference in mortality between the sexes

	♂	♀	♂ ratio (± *SD*)	Total	*p*‐Value
(a) Sex ratio					
Laying	109	101	0.519 (0.21)	210	.629
Embryo development	73	100	0.422 (0.27)	173	**.048**
Hatching	55	81	0.404 (0.35)	136	**.032**
Fledging	51	78	0.395 (0.36)	129	**.022**
(b) The stages of mortality					
Unfertilized eggs	‐	‐	‐	8	‐
Fertilized but undeveloped eggs	36 (33.03)	1 (0.99)	‐	37	‐
Developed but unhatched eggs	18 (24.66)	19 (19.00)	‐	37	‐
Hatched but died before fledging	4 (7.27)	3 (3.70)	‐	7	‐

Significant biases are shown in bold. Total sample size was 218 eggs from 41 clutches.

More than 80% of undeveloped eggs appeared to be fertilized but stopped developing at the early embryo stage, as a large number of nuclei stained with Hoechst 33342 were observed (82.1%; 23/28 eggs, Figure 1b). Conversely, 17.9% of the eggs with no nuclei or a small number of nuclei were considered unfertilized or fertilized but development stopped at around the 8‐cell stage (5/28 eggs, Figure 1c).

### 3.2 Offspring sex ratio

**Figure 2:**
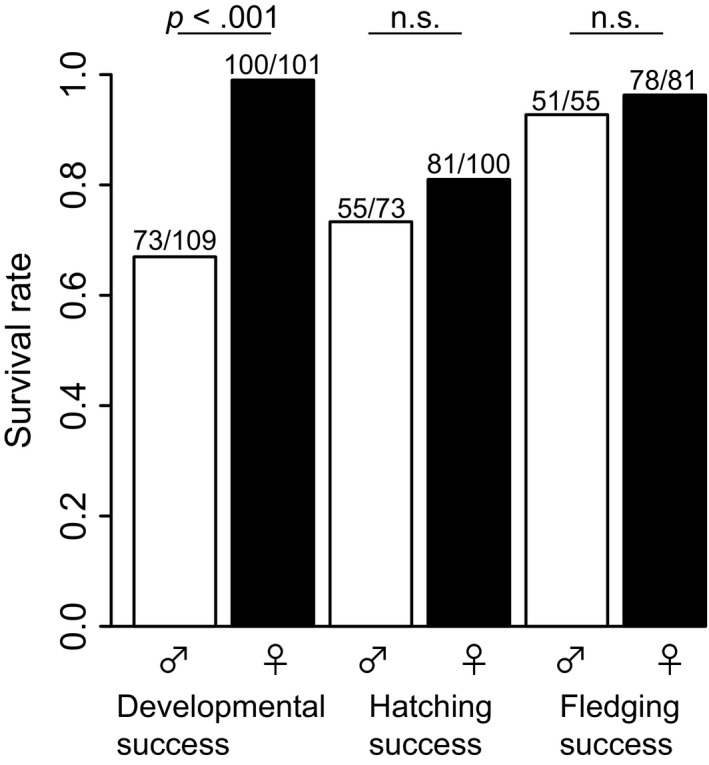
Survival rate of male and female offspring at three stages (developmental success, hatching success, and fledging success)

The secondary sex ratio was female‐biased due to SSM of male embryos, although the primary sex ratio was not biased to either sex (Table 1a, b). SSM of males was significantly higher than females at the embryo development stage (Figure 2, Tables 1b and 2). However, there was no significant difference in mortality between the sexes when developed embryos hatched or when chicks reached the fledging stage, except that survival rate during the hatching stage decreased significantly as the season progressed (Figure 2, Table 2).

**Table 2:** Analysis of surviving offspring and their sexes at three different growth stages

	*b*	*SE*	χ^*2*^	*df*	*p*
Embryo development success					
Intercept	5.926	1.240			
**Offspring sex**	−4.883	1.157	53.020	1	**<.001**
First day of egg laying	0.016	0.016	1.244	1	.265
Hatching success					
Intercept	0.311	0.010			
Offspring sex	−0.417	0.400	1.090	1	.297
**First day of egg laying**	1.347	0.004	6.646	1	**.010**
Fledging success					
Intercept	2.914	0.388			
Offspring sex	−0.001	0.785	0.835	1	.361
First day of egg laying	−0.713	0.785	0.003	1	.958

The variables in bold were selected for the final model.

### 3.3 The cause of embryo development failure

**Figure 3:**
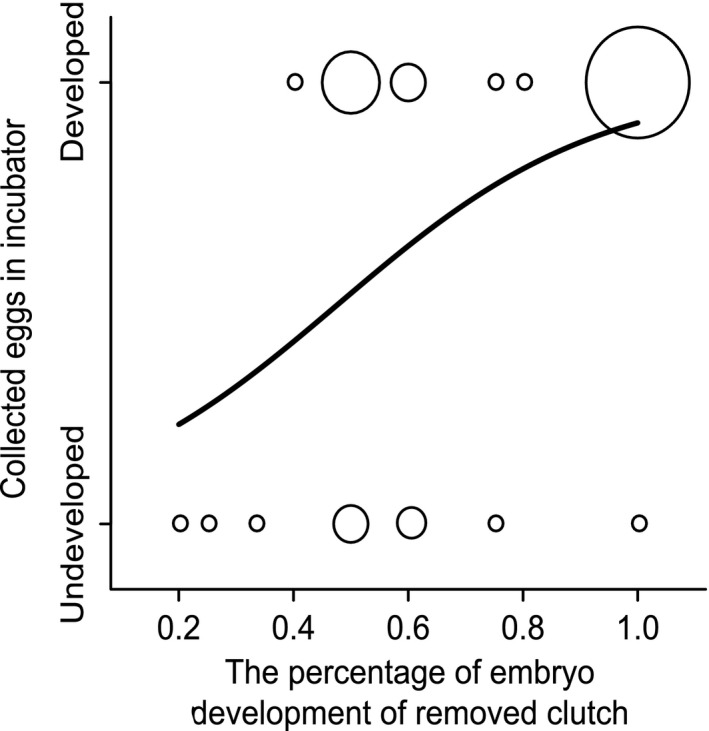
Relation between the embryo development/failure of eggs in an incubator and the percentage of embryo development in the original clutch. Circle size indicates sample size (from 1 to 9)

The incubation experiment supported the hypothesis that the cause of embryo development failure was lack of developmental ability in the egg. Developmental success in a collected egg and the percentage of development in the original clutch were positively correlated (*b* ± *SE* = 4.41 ± 2.02, *df* = 1, χ^2^ = −0.26, *p* = .03, Figure 3). In other words, an egg collected from a clutch with a low percentage of development was less likely to develop into an embryo in the incubator. Seasonality and egg‐laying order were excluded from the final model (seasonality: *b* ± *SE* = −0.01 ± 0.02, *df* = 1, χ^2^ = −0.26, *p* = .61; egg‐laying order: *b* ± *SE* = −0.28 ± 0.57, *df* = 1, χ^2^ = −6.38, *p* = .62).

## 4 DISCUSSION

This is the first report of offspring sex ratios at multiple stages, from egg laying to fledging, in a wild avian species. Offspring sex ratio was not biased at the primary stage (egg laying) but was female‐biased at the secondary sex ratio stage (from embryo development to fledging) due to SSM of male embryos during embryo development. Parental sex allocation has usually been studied at two stages, primary (at conception) and secondary (at birth), although these stages have not been clearly defined (West, 2009). Many previous studies have investigated primary and secondary sex ratio after the hatching stage (Aslam, Groothuis, Smits, & Woelders, 2014). In avian species, the definition of primary and secondary sex ratio has not been consistent across studies. For example, primary sex ratio can refer to the ratio at fertilization, (Aslam et al., 2014; DuRant et al., 2016; Eiby et al., 2008; Krackow, 2002), at laying (Alonso‐Alvarez, 2006; Czyż, Rowiński, & Wesołowski, 2012; Szász, Kiss, & Rosivall, 2012), or in the egg (Donald, 2007; Riordan, Lukacs, Huyvaert, & Dreitz, 2015). Secondary sex ratio can refer to the ratio after laying (DuRant et al., 2016; Szász et al., 2012), after hatching (Kilner, 1998; Riordan et al., 2015), at hatching (Eiby et al., 2008; Saunders & Cuthbert, 2015), at the chick stage (Czyż et al., 2012; Donald, 2007), or at fledging (Alonso‐Alvarez, 2006; Romano, Ambrosini, Caprioli, Bonisoli‐Alquati, & Saino, 2012), although there are distinct stages associated with the secondary sex ratio (i.e., embryo development, hatching, and fledging). To avoid semantic confusion on the difference of definition across studies, we need to define offspring sex ratio with respect to the major growth stages, which are at clutch, during incubation (embryo development), at hatching, and at fledging. As we show, ambiguous definition of the stages leads to incorrect calculation of offspring sex ratio. In addition, our results indicated the importance of tracing the successive changes in offspring sex ratio to detect the stage at which sex ratio becomes biased. Hence, a clear definition of primary and secondary offspring sex ratios helps us to understand the proximate mechanisms that bias the offspring sex ratio.

Our incubation experiment separated the potential causes of embryo developmental failure. Most of the unhatched eggs did not show any sign of embryo development, even though they were fertilized. These results concur with previous studies (Birkhead et al., 2008; Svensson et al., 2007). The high mortality of male embryos has also been reported in a Swedish population of Eurasian tree sparrows (Svensson et al., 2007). However, in the Swedish study, the authors sexed offspring from eggs that showed embryo development but did not hatch. In our study, male offspring that showed embryo development but did not hatch showed higher mortality than female offspring, although the difference was not statistically significant (Table 2). Previous studies indicated that SSM of embryos can be caused by the lack of developmental ability in the egg (Hemmings & Birkhead, 2015; Love et al., 2005; Navara, 2013; Rubolini et al., 2006; Rutkowska & Cichoń, 2006; von Engelhardt et al., 2004) or parental incubation (DuRant et al., 2016; Eiby et al., 2008). We showed that embryo developmental failure was caused by lack of developmental ability in the egg, rather than parental incubation. Though we did not identify sexes of offspring, our experiment strongly implied that SSM was also caused by lack of developmental ability in the egg because most of the undeveloped eggs were male (Table 2). This suggested that lethal genetic factors or hormonal factors impaired embryo development. For instance, previous studies hypothesized that the different concentration of Z‐chromosomal gene products in male (ZZ) and female (ZW) embryos resulted in SSM (Chandra, 1991; Krackow, 1999). Previous studies have provided mixed results of a hormonal effect, with maternally derived steroid hormones both increasing and decreasing embryo SSM (Love et al., 2005; Rubolini et al., 2006; Rutkowska & Cichoń, 2006; von Engelhardt et al., 2004). Furthermore, low number of sperm at fertilization was identified as one of the causes of early embryo death, although it was unknown whether the low number of sperm affected SSM of the embryo (Hemmings & Birkhead, 2015). Our results showed that embryos stopped normal development at around the 8‐cell stage, as there were granule‐like structures formed in the nuclei (Figure 1c). This suggests the occurrence of apoptosis and/or abnormal proteasome/autophagy systems (Tsukamoto et al., 2008). Reduced activation of autophagy is one of the candidate causes of early embryo death, but further analyses are required to clarify how it affects SSM.

To understand the evolution of SSM, we should examine the relation between SSM and environmental factors and life history. Recent meta‐analytical studies in birds found slight, but significant, biases in primary sex ratio in response to biological and temporal traits (Cassey, Ewen, & Møller, 2006) and mate attractiveness (Booksmythe, Mautz, Davis, Nakagawa, & Jennions, 2015). As we show, the secondary sex ratio does not always reflect the primary sex ratio because of effects such as SSM. As SSM may be a means of offspring sex ratio adjustment, the sex ratio bias should be determined across multiple stages of development in future studies.

## CONFLICT OF INTEREST

None declared.

## AUTHOR CONTRIBUTION

TK drafted the manuscript, conceived the study, and carried out the design of the study, the fieldwork, molecular laboratory work and statistical analyses; SM participated in the fieldwork and discussion; YT and HT participated in molecular laboratory work; SH helped the fieldwork; SK, GM, OKM, and KU participated in discussion; NK participated in the statistical analyses and discussion. All authors helped draft the manuscript and gave final approval for publication.
