# Supplementary material for: Male‐specific mortality biases secondary sex ratio in Eurasian tree sparrows Passer montanus
Source: Ecol Evol. 2017 Nov 6;7(24):10675–82. doi: 10.1002/ece3.3575 (PMC5743541; doi:10.1002/ece3.3575)
Supplement: Supplementary file 1 [file ECE3-7-10675-s001.docx]

**Data S1: Sex determination for germinal disks**

In the method by (Griffiths *et al.* 1998; Fridolfsson & Ellegren 1999), males showed one type of band (PCR products from Z-chromosome), whereas females showed two types of band (PCR products from Z- and W-chromosome) in agarose gel. This is a general method of sex determination in avian studies, but might lead wrong sex estimation because of contamination of paternal or maternal DNA (Arnold *et al.* 2003). This method does not mistake fertilized egg such that female or male eggs were judged as male or female respectively. However, we must take account following two points. One is that unfertilized egg possibly might have been sexed as male because residual sperms including Z-chromosome (Birkhead *et al.* 2008). In order to remove sperm and other substances, we washed outer side of vitelline membrane before staining nuclei. Furthermore, because we also showed that over 80% of undeveloped eggs were fertilized, we were less likely to mistake unfertilized eggs for male. The other is that maternal DNA contamination resulted from granulosa cells including Z and W-chromosome might lead to mistake male for female, or unfertilized egg for female. However, this contamination was not so important because the sex of undeveloped eggs was 36 males and 1 female in our investigation (Table 2b). Although it was possible that this one female was mistaken for male or unfertilized egg, this did not affect the entire tendency. Since we could observe the thousands of nuclei on the germinal discs of fertilized eggs (Fig. 1b), the amount of DNA from these nuclei would be enough to do PCR amplification.

**Reference**

Arnold, K.E., Orr, K.J., Griffiths, R. (2003) Primary sex ratios in birds: problems with molecular sex identification of undeveloped eggs. *Mol. Ecol*. **12**: 3451–3458.

Birkhead, T.R., Hall, J., Schut, E., Hemmings, N. (2008) Unhatched eggs: methods for discriminating between infertility and early embryo mortality. *Ibis*. **150**: 508–517.

Fridolfsson, A.K. & Ellegren, H. (1999) A Simple and universal method for molecular sexing of non-ratite birds. *J. Avian. Biol*. **30**: 116–121.

Griffiths, R., Double, M.C., Orr, K., Dawson, R.J.G. (1998) A DNA test to sex most birds. *Mol. Ecol.* **7**: 1071–1075.
